# Supplementary material for: Benzalkonium Chloride, Even at Low Concentrations, Deteriorates Intracellular Metabolic Capacity in Human Conjunctival Fibroblasts
Source: Biomedicines. 2022 Sep 18;10(9):2315. doi: 10.3390/biomedicines10092315 (PMC9496331; doi:10.3390/biomedicines10092315)
Supplement: Supplementary file 1 [file biomedicines-10-02315-s001.zip › Supplemental Figure S1.pdf]

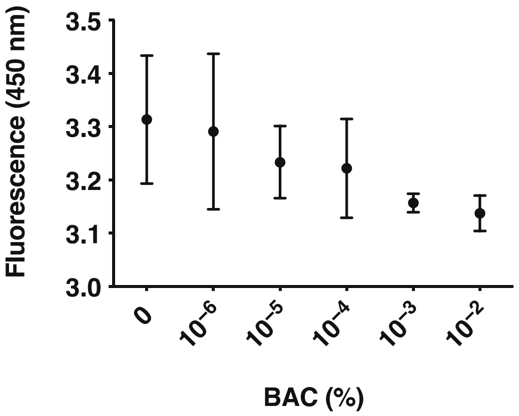

Supplemental Figure. S1. Cytotoxicity assay To estimate the cytotoxic effects of BAC at different concentrations (0, 10−6 % ~ 10−2 %) on 2D cultured HconF cells, the survival of WST-8 Cells was evaluated by means of a Cell Counting Kit-8 (Dojindo, Tokyo Japan) according to the manufacturer protocol. Briefly, after culturing 2D HconF cells (5000 cells/well) for 24 hrs, they were incubated with 10 ◎l of reactive solution for 4 hrs. The absorbance of each cell at 450 nm was measured using a microplate reader (multimode plate reader EnSpire®, PerkinElmer, MA U.S.A.) and plotted (n=4). Data are expressed; the mean ± the standard error of the mean (SEM). *P<0.05; ANOVA followed by a Tukey’s multiple comparison test.
