# Supplementary material for: Benzalkonium Chloride, Even at Low Concentrations, Deteriorates Intracellular Metabolic Capacity in Human Conjunctival Fibroblasts
Source: Biomedicines. 2022 Sep 18;10(9):2315. doi: 10.3390/biomedicines10092315 (PMC9496331; doi:10.3390/biomedicines10092315)
Supplement: Supplementary file 1 [file biomedicines-10-02315-s001.zip › Supplemental Table S1 primers.pdf]

Supplemental Table S1 Sequences of primers of qPCR

		Sequence	Exon Location	RefSeq Number
human RPLP0*1	Probe	5'-/56-FAM/CCCTGTCTT/ZEN/CCCTGGGCATCAC/3IABkFQ/-3'	2-3	NM_001002
	Primer2	5'-TCGTCTTTAAACCCTGCGTG-3'		
	Primer1	5'-TGTCTGCTCCCACAATGAAAC-3'		
human COL1A1*1	Probe	5'-/56-FAM/TCGAGGGCC/ZEN/AAGACGAAGACATC/3IABkFQ/-3'	1-2	NM_000088
	Primer2	5'-GACATGTTCAGCTTTGTGGAC-3'		
	Primer1	5'-TTCTGTACGCAGGTGATTGG-3'		
human COL4A1*1	Probe	5′-/56-FAM/TCATACAGA/ZEN/CTTGGCAGCGGCT/3IABkFQ/-3′	51-52	NM_001845
	Primer2	5′-AGAGAGGAGCGAGATGTTCA-3′		
	Primer1	5′-TGAGTCAGGCTTCATTATGTTCT-3′		
human COL6A1*1	Probe	5′-/56-FAM/CAGGTTTCG/ZEN/GTCACAGCGGTAGT/3IABkFQ/-3′	2-3	NM_001848
	Primer2	5'-CCTCGTGGACAAAGTCAAGT-3'		
	Primer1	5'-GTGAGGCCTTGGATGATCTC-3'		
human FN1*1	Probe	5′-/56-FAM/TACAGCTTA/ZEN/TTCTCCCTCGCCCAG/3IABkFQ/-3′	3-4	NM_212482
	Primer2	5'-CGTCCTAAAGACTCCATGATCTG-3'		
	Primer1	5'-ACCAATCTTGTAGGACTGACC-3'		
human αSMA*1	Probe	5'-/56-FAM/AGACCCTGT/ZEN/TCCAGCCATCCTTC/3IABkFQ/-3'	8-9	NM_001613
	Primer2	5'-AGAGTTACGAGTTGCCTGATG-3'		
	Primer1	5'-CTGTTGTAGGTGGTTTCATGGA-3'		
human Grp78*2	Forward	5'-CATCACGCCGTCCTATGTCG-3'		NM_005347
	Reverse	5'-CGTCAAAGACCGTGTTCTCG-3'		
human GRP94*2	Forward	5'-CTGGGACTGGGAACTTATGAATG-3'		NM_003299
	Reverse	5'-TCCATATTCGTCAAACAGACCAC-3'		
human XBP*2	Forward	5'-AGTAGCAGCTCAGACTGCCA-3'		NM_005080
	Reverse	5'-CCTGGTTCTCAACTACAAGGC-3'		
human sXBP*2	Forward	5'-GGTCTGCTGAGTCCGCAGCAGG-3'		AB076384
	Reverse	5'-GGGCTTGGTATATATGTGG-3'		
human CHOP*2	Forward	5'-GGAGAACCAGGAAACGGAAAC-3'		NM_004083
	Reverse	5'-TCTCCTTCATGCGCTGCTTT-3'		
Human PGC1 α *1	Probe	5'-/56-FAM/ ACCAGCCTC/ZEN/ TTTGCCCAGATCTTC/-3'	1-2	NM_013261
	Primer2	5'- TGTCTGTATCCAAGTCGTTCAC-3'		
	Primer1	5'- GAGTCTGTTATGGAGTGACATCG -3'		
Human HIF1A*1	Probe	5'-/56-FAM/TGGCAAGCA/ZEN/TCCTGTACTGTCCTG/3IABkFQ/-3'	8-9	NM_181054
	Primer2	5'-CAACCCAGACATATCCACCTC-3'		
	Primer1	5'-CTCTGATCATCTGACCAAAACTCA -3'		

*1 Taqman probes (IDT, Coralville, IA, USA). *2 SYBR probes (IDT, Coralville, IA, USA).
